# Lomitapide response in a cohort of patients with homozygous familial hypercholesterolemia and the potential influence of *MTTP* gene variants

**DOI:** 10.1186/s13023-025-04033-3

**Published:** 2025-10-14

**Authors:** Genovefa Kolovou, Vana Kolovou, Katherine Anagnostopoulou, Georgia Anastasiou, Petros Kalogeropoulos, Evangelos Liberopoulos

**Affiliations:** 1 Cardiometabolic Center, Lipoprotein Apheresis and Lipid Disorders Clinic, Metropolitan Hospital,9 Ethn. Makariou & 1 El Venizelou Str, N Faliro, 185 47 Piraeus, Greece; 2 Molecular Genetics Department, Genomedica S.A, Piraeus, Greece; 3 2nd Department of Internal MedicineSchool of Medicine, University of Ioannina, Ioannina, Greece; 4 1st Department of Propedeutic MedicineSchool of Medicine, National and Kapodistrian University of Athens, Athens, Greece

**Keywords:** Homozygous familial hypercholesterolemia, Lomitapide, MTP, *MTTP*, LDLR, LDL-C, Lipoprotein metabolism, Genetic variants

## Abstract

**Background:**

Homozygous familial hypercholesterolemia (HoFH) is a rare inherited disorder of lipoprotein metabolism caused by pathogenic variants in both alleles of key low-density lipoprotein receptor (LDLR)-mediated pathway genes, resulting in very high LDL cholesterol (LDL-C) levels from birth. The microsomal triglyceride transfer protein (MTP) inhibitor lomitapide, is an effective treatment for lowering LDL-C in HoFH that acts independently of LDLR. This study investigated the response to lomitapide treatment and the potential impact of *MTTP* gene variants in a cohort of patients with HoFH.

**Methods:**

Data were extracted from medical records of patients diagnosed with HoFH and receiving treatment with lomitapide in addition to background statin + ezetimibe + PCSK9 inhibitor therapy. Data on LDL-C levels before and after lomitapide treatment were collected from patient medical histories. Genetic sequencing of all exonic and intronic flanking regions of the *MTTP* gene was carried out for all patients with genomic DNA isolated from whole blood.

**Results:**

A total of 13 patients with a diagnosis of HoFH were identified (mean ± standard deviation age, 47.3 ± 17.3 years). The median (range) dose of lomitapide was 20 mg/day (10 to 60 mg/day). Median (range) baseline LDL-C (before lomitapide) was 240 mg/dL (162 to 478 mg/dL). Following lomitapide treatment the median (range) LDL-C level was 119 mg/dL (56 to 305 mg/dL), and all patients reported a reduction in LDL-C with lomitapide. A total of 151 *MTTP* gene variants were identified encompassing 50 distinct variants. There was a trend for more variants per patient with LDL-C reduction > 50% vs. patients with LDL-C reduction ≤ 50% (difference, 8.5; 95% confidence interval [CI] − 1.2, 18.1; *P* = 0.08). Several *MTTP* gene variants (rs17533489, rs79194015, rs745075, rs41275715, rs1491246, and rs17533517) previously identified as potentially associated with a greater response to lomitapide treatment were significantly more common in patients with a reduction in LDL-C > 50% than those with a reduction in LDL-C ≤ 50% (difference, 3.9; 95% CI 3.3, 4.5; *P* < 0.001).

**Conclusions:**

This study builds upon previous findings by our group suggesting that variants in the *MTTP* gene may influence response to lomitapide. This study further presented a number of variants that may be uniquely associated with higher or lower response to lomitapide treatment.

**Supplementary Information:**

The online version contains supplementary material available at 10.1186/s13023-025-04033-3.

## Background

Homozygous familial hypercholesterolemia (HoFH) is an ultra-rare inherited disorder of lipoprotein metabolism that is typically caused by biallelic autosomal semi-dominant pathogenic variants in key genes mediating low-density lipoprotein receptor (*LDLR*) function [1–3]. Additionally, phenotypic HoFH occurs when monoallelic pathogenic variants are inherited in two different genes [4] These variants result in very high LDL cholesterol (LDL-C) levels from birth; in some cases, these are up to ten times the level of those without HoFH [1, 3]. Patients have an extremely high risk of cardiovascular disease (CVD) due to accelerated development of atherosclerosis [5, 6]. Interventions to reduce elevated levels of LDL-C must, therefore, be initiated as early as possible to manage CVD risk and prevent premature death [5, 6]. Nonpharmacological intervention, in the form of lifestyle modification (primarily institution of low-fat diet or cessation of smoking where relevant) has an important role in the treatment of HoFH; however, this alone is not sufficient to prevent the adverse and premature progression of CVD [5, 7]. Thus, pharmacological interventions are required to lower LDL-C [5]; high-intensity statins, such as rosuvastatin or atorvastatin, and ezetimibe are usually the first therapies that are introduced [8]. These treatments are rarely sufficient to lower LDL-C below targets levels, necessitating the introduction of further lipid-lowering interventions, such as lipoprotein apheresis and proprotein convertase subtilisin kexin type-9 (PSCK9) inhibitors [2, 9]. Additionally, there are treatments that work independently of LDLR, such as the microsomal triglyceride transfer protein (MTP) inhibitor lomitapide and the recently approved angiopoetin-like 3 inhibitor, evinacumab [8–10]. If intensive lipid-lowering is not initiated in early childhood, there is a high risk of severe cardiovascular morbidity and mortality [11, 12].

Lomitapide is an effective treatment for lowering LDL-C in HoFH with the advantage of acting independently of LDLR [8], whereas the effect of most other pharmacological interventions is dependent on residual LDLR function [2, 13]. Nevertheless, individual response to lomitapide can vary, with some patients requiring a higher lomitapide dose for equivalent LDL-C reduction than others [8]; a better understanding of factors that impact the effectiveness of lomitapide could assist in the personalized management of HoFH. The target of lomitapide, MTP, plays a crucial role in production and secretion of apolipoprotein B (apoB)-containing lipoproteins (very-low-density lipoproteins and chylomicrons) in the liver and intestines, respectively [14, 15]. Genetic variations in the *MTTP* gene caused by single nucleotide polymorphisms (SNPs) affect the lipid transfer activity of MTP [16]. A previous case series hypothesized that genetic variants in the *MTTP* gene encoding MTP may influence HoFH patients’ response to MTP inhibitors such as lomitapide [17]. This study further investigated inter-patient variability in response to lomitapide and the potential impact of *MTTP* gene variants on treatment response.

## Methods

### Study subjects

Data were extracted from medical records of 13 patients diagnosed with HoFH and receiving treatment with lomitapide in addition to background therapy with maximal tolerated dose of statin + ezetimibe 10 mg daily and PCSK9 inhibitors. Concomitant therapies were continued without any changes throughout the entire study and adherence to background treatment was satisfied. If patients were receiving lipoprotein apheresis, sessions were stopped one month before lomitapide was added and the lipid values of at least one month after stopping lipoprotein apheresis sessions were included. Data were collected from patient medical histories including CVD history, liver ultrasound results and HoFH pathogenic variants, and LDL-C levels before and after lomitapide treatment, i.e., at the most recent follow-up visit. Genetic sequencing of all exonic and intronic flanking regions of the *MTTP* gene was carried out for all patients with genomic DNA isolated from whole blood as previously described [17].

### Data analysis

The total number of different *MTTP* variants in the full cohort was calculated. The National Center for Biotechnology Information (NCBI) database of SNPs (dbSNP) and ClinVar databases were queried for individual variant information. Variants were merged where this had been applied on NCBI dbSNP. *MTTP* variants were stratified into different LDL-C reduction subgroups, using similar stratification methods to a previous study performed by our group [17]. Patients were stratified by whether lomitapide treatment induced a reduction in LDL-C of ≤ 50% or > 50%. The rationale for choosing the cut-off of 50% LDL-C reduction is based on the average reduction in LDL-C in lomitapide studies [2]. As an additional exploratory analysis, patients were further stratified by whether lomitapide induced a reduction in LDL-C of ≤ 25%. As per our previous study [17], variants unique to the different subgroups were identified. Variants were then sorted into these subgroups to determine if any were uniquely associated with a high or lower response to lomitapide treatment.

All statistical analyses compared between the group with a ≤ 50% reduction in LDL-C and the group with a > 50% reduction in LDL-C. Individual variants were compared between groups using Fisher’s exact test. Continuous variables were compared between groups using the unpaired t-test if found to be normally distributed or the Mann-Whitney test if not found to be normally distributed.

## Results

Patient histories and LDL-C levels before and after lomitapide treatment are shown in Table 1. Six patients were on lipoprotein apheresis sessions biweekly prior to initiation of lomitapide. The adherence to background treatment was satisfied. All patients had received lomitapide for at least 2 years at the time of data collection and dosage had been up-titrated based on LDL-C treatment target achievement and tolerability. A total of 13 patients with a diagnosis of HoFH were identified, with a mean ± standard deviation age 47.3 ± 17.3 years (median [range]: 46 [22 to 75] years). CVD history was available for 11/13 patients, and included myocardial infarction, coronary artery bypass graft, aortic valve replacement, percutaneous coronary intervention, carotid stenosis, and severe heart failure.HoFH genetic data were available for 11/13 patients, with all but one showing bi-allelic mutations in the *LDLR* gene, with the remaining patient showing a bi-allelic mutation in the *APOB* gene. Median (range) baseline LDL-C level prior to any lipid-lowering treatment was reported for 11/13 patients and was 780 mg/dL (240 to 1000 mg/dL). Median (range) baseline LDL-C before lomitapide was 240 mg/dL (162 to 478 mg/dL), with data available for all patients. Concomitant medications other than lipid-lowering treatment during the period of lomitapide treatment are shown in Supplementary Table 1, Additional File 1.

**Table 1:** Patient histories and LDL-C levels before and after lomitapide treatment

Patient ID	Age (years)	CVD history		Steatosis grading (liver ultrasound)^a^	HoFH genetics^b^	Lomitapide dose (mg/day)	Lomitapide duration (years)	LDL-C (mg/dL)			
								At diagnosis	Baseline on LLT	After lomitapide	% change from baseline LLT to after lomitapide
		Before lomitapide	After lomitapide								
> 50% LDL-C reduction from baseline LLT to after lomitapide											
9	75	Non-fatal MI (NSTEMI RCA), non-fatal MI (strain)	Non-fatal MI (NSTEMI RCA-PCI)	N/A	*LDLR*: 1775G > A	10	5	N/A	197	56	–71.6
11	29	CABG, AVR, carotid stenosis	–	0	*LDLR*: c.1285G > A (p.Val429Met)	20	9	950	330	114	–65.5
6	46	–	–	1	*LDLR*: c.166T > C; p.Ser56Pro and c.1285G > A; p.Val429Met	30	7	511	478	185	–61.3
5	42	Non-fatal MI, PCI	–	1	*LDLR*: c.81 C > G; p.Cys27Trp and c.1646G > A; p.Gly549Asp	40	4	371	235	106	–54.8
13	61	CABG x2, PCI x2, AVR, carotid endarterectomy, severe heart failure	PCI, death	1	N/A	20	6	780	259	118	–54.4
7	42	CABG	angina	N/A	*LDLR*: p.Gly1775Ala	10	4.5	N/A	162	74	–54.3
≤ 50% LDL-C reduction from baseline LLT to after lomitapide											
3	62	Non-fatal MI, CABG, carotid stenosis	–	1	*LDLR*: c.858 C > A; p.Ser286Arg and c.1291G > A; p.Ala431Thr	10	2	240	176	99	–43.8
4	53	Non-fatal MI, PCI, carotid endarterectomy, severe heart failure	–	0	*LDLR*: c.858 C > A; p.Ser286Arg and c.1291G > A; p.Ala431Thr	10	2	500	204	119	–41.7
1	22	Mild aortic valve stenosis	–	0	*LDLR*: c.666 C > A; p.Cys222* and c.1646G > A; p.Gly549Asp	60	4	1000	350	220	–37.1
10	32	Mild carotid stenosis	–	0	*LDLR*: c.1448G > A, (p.Trp483X) and c.1646G > A	20	9	900	472	305	–35.4
≤ 25% LDL-C reduction from baseline LLT to after lomitapide											
2	69	Mild carotid stenosis	–	0	*APOB*: c.10580G > A; p.Arg3527Gln (rs5742904) Het	40	2	600	240	180	–25.0
8	24	–	–	0	*LDLR*: G1646A	20	5	987	243	188	–22.6
12	58	CABG + AVR, carotid endarterectomy, severe heart failure	–	1	N/A	20	6	900	220	196	–10.9

^a^Liver ultrasound was recorded as part of initial diagnostic work; ^b^All patients had two copies of each mutation listed

*APOB*, apolipoprotein B-100; AVR, aortic valve replacement; CABG, coronary artery bypass graft; CVD, cardiovascular disease; ID, identification; LDL-C, low-density lipoprotein cholesterol; *LDLR*, low-density lipoprotein receptor; LLT, lipid-lowering treatment; MI, myocardial infarction; N/A, not available; NSTEMI, non-ST segment elevation myocardial infarction; PCI, percutaneous coronary intervention; RCA, right coronary artery

At the latest follow-up following lomitapide treatment, the median (range) LDL-C level was 119 mg/dL (56 to 305 mg/dL). All patients reported a reduction in LDL-C with lomitapide. The median (range) percentage change in LDL-C levels after lomitapide treatment was − 43.8% (–71.6 to − 10.9%) with a median (range) dose of lomitapide 20 mg/day (10 to 60 mg/day). In total, 3 patients achieved an LDL-C level of < 100 mg/dL after lomitapide treatment (Table 1). One patient achieved an LDL-C level < 70 mg/dL after lomitapide treatment (patient 9, 56 mg/dL; LDL-C change, − 71.6%), and another patient an LDL-C of 74 mg/dL (patient 7; LDL-C change, − 54.3%).

Patients were stratified according to change of LDL-C reduction following lomitapide (Table 2). In response to lomitapide treatment, 6/13 patients (46.2%) showed a reduction in LDL-C > 50%, while 7/13 (53.8%) showed reductions of LDL-C ≤ 50%. Median dose of lomitapide was the same for both the LDL-C > 50% and ≤ 50% groups (20 mg/day); however, the maximum dose was higher in the LDL-C ≤ 50% reduction group (60 mg/day) compared with the LDL-C > 50% reduction group (40 mg/day).

**Table 2:** Stratification according to LDL-C reduction after lomitapide

Category	Number
Total patients	13
Number of patients with change in LDL-C > 50%	6
Number of patients with change in LDL-C ≤ 50%^a^	7
Number of patients with change in LDL-C ≤ 25%	3
Total number of *MTTP* variants in full cohort	151
Number of variants in LDL-C > 50% reduction subgroup	97
Number of variants LDL-C ≤ 50% reduction subgroup^a^	54
Number of variants LDL-C ≤ 25% reduction subgroup	16
Total number of distinct *MTTP* variants in full cohort	50
Number of variants unique to LDL-C > 50% reduction subgroup	15
Number of variants in LDL-C ≤ 50% reduction subgroup but not in LDL-C > 50% reduction subgroup^a^	8
Number of variants in LDL ≤ 25% reduction subgroup not observed in patients with > 25% LDL-C reduction	2

^a^Includes variants in LDL-C ≤ 25% reduction subgroup, low-density lipoprotein cholesterol

LDL-C, low-density lipoprotein cholesterol

A total of 151 *MTTP* variants were identified encompassing 50 distinct variants. Querying of dbSNP revealed four variants associated with insertion/deletion of nucleotide sequences or nucleotide substitution leading to a synonymous variant (rs545365701/rs756785244/rs757684569, rs375598284, rs397878861/rs5860586 and rs982424). These variants appeared in 4/6 patients with reduction in LDL-C > 50% and 6/7 patients with reduction in LDL-C ≤ 50% and did not correlate with an overall influence on LDL-C reduction with lomitapide. Variants unique to either LDL-C > 50% or ≤ 50% reduction groups – and therefore potentially associated with lomitapide response – are listed in Table 3. Two variants associated with insertion/deletions (rs375598284 and rs397878861/rs5860586) were unique to patients in the LDL-C > 50% reduction group, suggesting that these variants did not impair the lipid-lowering effect of lomitapide in these patients, or alternatively may be advantageous. A single variant, rs982424, identified as a synonymous variant, was only present in two patients with a reduction in LDL-C ≤ 50%: patient 1 (37.1% reduction) and patient 12 (10.9% reduction; lowest responder in the cohort).

**Table 3:** Variants unique to the LDL-C > 50% and ≤ 50% reduction groups

LDL-C > 50% reduction	LDL-C ≤ 50% reduction
rs112407688, rs112568939, rs113337987, rs113557405, rs113653192, rs114681504, rs141186441, rs1491244, rs17533489, rs375598284^a^, rs397878861/rs5860586^a^, rs61733139, rs6832119, rs6832927 and rs74542928	rs11944749, rs17532601, rs2255119, rs2306984, rs2306986^b^, rs3792683^b^, rs7533489, rs982424^c^, rs114681504 and rs17533489

^a^Associated with insertion/deletion; ^b^associated with patients who experienced a very low response (≤ 25%); ^c^associated with stop codon insertion

In a previous study [17], we showed that 6 identified variants (rs17533489, rs79194015, rs745075, rs41275715, rs1491246, rs17533517) were associated with an enhanced effect of lomitapide (reduction in LDL-C > 50%). All of these variants were observed in the present study cohort and generally occurred in patients with reduction in LDL-C > 50%. The only occurrence of these variants in patients with reduction in LDL-C ≤ 50% was in patient 3 who was receiving treatment with 10 mg lomitapide. This patient had 5/6 of the variants and the greatest reduction in LDL-C in the ≤ 50% group (43.8%). A comparison of these variants between LDL-C > 50% and ≤ 50% reduction groups is shown in Table 4. The variant rs17533489 was significantly more common in the LDL-C > 50% reduction group (difference, 67%; 95% confidence interval [CI] 29%, 100%; *p* = 0.02) and there was also weak evidence that rs79194015, rs745075 and rs41275715 were more common in the LDL-C > 50% reduction group, although these results were not statistically significant; however, the overall occurrence of the 6 variants was found to be significantly greater in patients with reduction in LDL-C > 50% vs. the ≤ 50% reduction group (difference, 3.9; 95% CI 3.3, 4.5; *p* < 0.001). A further variant of interest previously identified by our group, rs3816873, did not significantly vary between the two groups (difference, − 29%; 95% CI − 62%, 5%; *p* = 0.46).

**Table 4:** Variant association with LDL-C > 50% and ≤ 50% reduction groups – statistical comparisons

Variable	Reduction in LDL-C > 50% (*n* = 6)	Reduction in LDL-C ≤ 50% (*n* = 7)	Difference (95% CI)	*P*-value
Number of variants per patient	16.2 ± 10.4	7.7 ± 4.8	8.5 (–1.2, 18.1)	0.08
rs982424	0 (0%)	2 (29%)	–29% (–62%, 5%)	0.46
Variants previously identified in patients with reduction in LDL-C > 50% [17]	4.6 ± 1.7	0.7 ± 1.8	3.9 (3.3, 4.5)	**< 0.001**
rs17533489	4 (67%)	0 (0%)	67% (29%, 100%)	**0.02**
rs79194015	4 (67%)	1 (14%)	52% (7%, 98%)	0.10
rs745075	4 (67%)	1 (14%)	52% (7%, 98%)	0.10
rs41275715	4 (67%)	1 (14%)	52% (7%, 98%)	0.10
rs1491246	2 (33%)	1 (14%)	19% (-27%, 65%)	0.56
rs17533517	3 (50%)	1 (14%)	36% (-12%, 83%)	0.27
Previously identified variant of interest [17]				
rs3816873	4 (67%)	3 (43%)	24% (-29%, 76%)	0.59

Summary statistics are mean ± standard deviation or mean (percentage). Significance was determined by *p* < 0.05

CI, confidence interval; LDL-C, low-density lipoprotein cholesterol

**Fig. 1:**
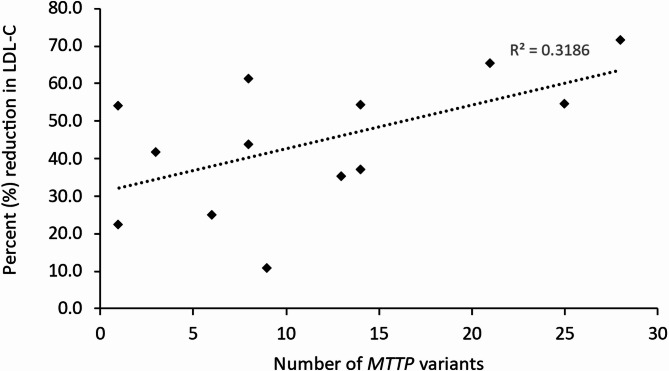
Correlation between *MTTP* variants observed in each patient and individual response to lomitapide LDL-C, low-density lipoprotein cholesterol; *MTTP*, microsomal triglyceride transfer protein

Although there was a similar number of patients in both LDL-C > 50% and ≤ 50% reduction groups in the present analysis (6/13 and 7/13 patients, respectively), there was approximately double the total number of variants in the former group than the latter group (Table 2). There was a mean of 16 variants per patient with LDL-C reduction > 50%, compared with a mean of 8 variants per patient with LDL-C reduction ≤ 50%, and this difference approached significance (difference, 8.5; 95% CI − 1.2, 18.1; *p* = 0.08; Table 4). There was a weak/moderate positive correlation between the number of *MTTP* variants observed in each patient and individual response to lomitapide (R^2^ = 0.3186; Fig. 1).

## Discussion

In this study, response to lomitapide treatment was assessed via the change in LDL-C levels in patients with HoFH. Patients possessed different SNPs in the *MTTP* gene, which our previous small study (*n* = 4) hypothesized would affect LDL-C reduction after lomitapide treatment, owing to the possible effect genetic variants may have on the mechanism of action of lomitapide as an inhibitor of MTP [17].

According to previous methodology [17], it could be argued that the unique variants identified in this patient cohort may be associated with the degree of response to lomitapide treatment. Of note in the present study is the rs982424 variant, which was only seen in patients with lower response (LDL-C reduction ≤ 50%). This variant does not alter amino acid sequence of the protein unlike other identified variants which affect non-coding regions of the gene and may regulate gene expression.

Interestingly, all the variants identified as potentially associated with reduction in LDL-C > 50% in our previous study were found in the present cohort and were determined to be significantly more common in the higher response group reported here. This supports the hypothesis that these variants are associated with greater response to lomitapide treatment; rs17533489 is associated with the strongest evidence for this as it is present in patients with the greatest reduction in LDL-C following lomitapide treatment. Another highlighted variant, rs745075, was found in a study of 202 statin-treated patients to be significantly associated with LDL-C lowering compared with patients without the variant (*p* = 0.035) [18]. Although low patient numbers in the present study potentially prevented a significant association between this variant and a high response, the findings presented here suggest that the variant may also be associated with lipid-lowering response to lomitapide.

In addition to some individual variants potentially being associated with reduction in LDL-C > 50%, there was evidence that the cumulative effect of multiple minor variants could also be associated with this, as a greater number of variants were observed in the LDL-C > 50% reduction group vs. the LDL-C ≤ 50% reduction group. This is further supported by the weak/moderate positive correlation between LDL-C reduction following lomitapide treatment and the number of *MTTP* variants found in each patient. A possible explanation for this may be that the cumulative effect of multiple minor genetic variants within the MTP protein causes aberrant function/secretion to an extent that lomitapide binds a higher proportion of MTP protein, thereby increasing the treatment effect.

We previously highlighted rs3816873 as a variant of interest [17], suggesting that this is a variant that confers reduced structural stability of MTP and decreased binding to apoB, and possibly associated with a higher risk of CVD through the overloading of triglycerides in the myocardium [19]. In this patient cohort there was no significant difference in this variant between the LDL-C > 50% and LDL-C ≤ 50% reduction groups; rs3816873 occurred in both patient groups.

Several other variants identified in this study have been reported as being associated with lipid dysfunction. Dai et al. [20]. reported that rs2306986 was associated with non-alcoholic fatty liver disease (NAFLD) in obese children. It was concluded that the observed effect associated with this *MTTP* variant may alter lipid metabolism by disrupting the MTP protein function, leading to aberrant triglyceride storage in the liver. In the present study, rs2306986 was unique to the patient in LDL-C ≤ 50% reduction group with the smallest change in LDL-C levels, and this could be related to the above previously identified change in function. Furthermore, a cross-sectional study reported that rs2306986, along with rs3792683 and rs2306985, were associated with significantly lower serum triglycerides [21], with rs2306985 also associated with increased risk of NAFLD. Similar to rs2306986, rs3792683 was uniquely identified in the LDL-C ≤ 50% reduction group in the present study.

The *MTTP* variant rs1800804 encodes a T > C alteration in the promoter region of the *MTTP* gene [22]. There is prior research to suggest that deletion from the region where this variant lies may increase promoter activity by 250% [23]; however, rs1800804 has previously been reported as not affecting lipid measurements or angiographically assessed coronary stenosis [22, 24, 25] suggesting it does not lead to increased MTP expression [22]. Both rs1800804 and rs1057613 have also been shown to be associated with NAFLD risk (increased and decreased risk, respectively) [14]; however, the current study identified these variants in both patient groups, suggesting that, as with rs3816873, they do not influence how lomitapide binds with MTP and response to lomitapide treatment is unaffected. While these variants may not directly affect response to lomitapide treatment, their presence may influence subsequent hepatic steatosis status. The liver ultrasound data available for this cohort of patients with HoFH receiving lomitapide treatment indicated that the highest liver steatosis grading was 1 (mild), and there was, therefore, little evidence that particular *MTTP* variants are contributing to steatosis in these patients prior to initiation of lomitapide treatment. It is recommended that in the event of liver-related AEs, such as hepatic steatosis or increased aminotransferase levels, patients either reduce their dose of lomitapide or temporarily suspend treatment. Dose reduction or suspension of treatment may then lead to an increase in LDL-C levels.

Lomitapide is a small-molecule that is metabolized by CYP3A4 in the liver [26, 27]. As such, lomitapide response is likely influenced by CYP3A4 enzyme activity, which can show significant inter-patient variability and can be impacted by commonly prescribed drugs in cardiovascular disease such as diltiazem or verapamil [28]. While CYP3A4 activity with concomitant medication was not accounted for in this study, the patient with the highest response to lomitapide was also receiving a low dose of amiodarone, a weak inhibitor of CYP3A4 [29, 30]. This may suggest that the higher response to lomitapide was partially aided by the inhibition of CYP3A4 and a reduction in metabolism of the small molecule; however, no other patients were receiving any concomitant medications that may have affected the activity of CYP3A4 (Supplementary Table 1, Additional File 1) and several were able to maintain high levels of LDL-C reduction. Of note, in the previous studies, our group examined, the relationship between polymorphic enzyme P450 oxidoreductase (POR)*28 polymorphism, which is associated with increased activity of CYP3A enzymes, and the response to atorvastatin and simvastatin, in 350 hyperlipidemic patients [31]. The POR*28 allele was not associated with the lipid-lowering effect of atorvastatin and the results were replicated in an independent simvastatin-treated population. We also analyzed inter-individual variability in relation to CYP3A4 intron 6 C > T polymorphism (CYP3A4*22 allele, rs35599367) and the response to atorvastatin and simvastatin [32]; there was no association with the lipid-lowering response to both drugs [33].

Despite the variability in lomitapide response seen in the present cohort, it is encouraging that the majority of patients (8/13) showed at least a 40% reduction in LDL-C subsequent to the introduction of lomitapide. It is also encouraging that the great majority of cardiovascular events were reported prior to lomitapide initiation; however, as only two patients fell under or approached the current European Atherosclerosis Society recommended target LDL-C level of < 70 mg/dL for adults without atherosclerotic CVD risk factors and no patient achieved the < 55 mg/dL for adults with atherosclerotic CVD or major atherosclerotic CVD risk factors [2], this highlights the challenge of achieving these levels in patients with HoFH, and the need for dosage up-titration or adding another drug such as evinacumab if available. Lipoprotein apheresis is also a valuable tool for achieving recommended target LDL-C level. Lipoprotein apheresis sessions remove about 60–70% of.

LDL-C, but the LDL-C reduction is transitory and associated with a rebound elevation in lipid levels after 7–14 days. In some cases, hybrid (all available drug treatment and lipoprotein apheresis) therapy may be needed.

There were several limitations to the present study. Firstly, there are the limitations inherent to any retrospective observational study with respect to the potential for bias. In addition, the study design meant that dosage of lomitapide was not standardized, while the changes in LDL-C attributed to the introduction of lomitapide were not time-averaged but were those assessed at the last follow-up. The data are therefore a ‘snapshot’ of response to lomitapide and could have shown variability across the course of treatment. As such, and with respect to the analysis of the influence of *MTTP* variants, patients could have fallen on either side of the 50% reduction threshold at different points in time between commencement of lomitapide treatment and last follow-up. An alternative way of conducting the analysis would be to compare the upper and lower quartiles of response; however, this approach would require a much larger sample size, which is very difficult for a rare disease as HoFH. The current study can only be considered as an extension of our previous hypothesis-generating study [17]. Further research, such as in vitro binding studies or computational modeling is required to confirm whether particular *MTTP* variants indeed abrogate or enhance the effectiveness of lomitapide. Further research could also examine the extent to which genetic variation in CYP3A4 influences response to lomitapide.

## Conclusions

Lomitapide can effectively reduce LDL-C in HoFH patients receiving background lipid-lowering therapy. Nevertheless, the response to treatment is variable. The present analysis lends support to previous findings by our group [17], suggesting that variants in the *MTTP* gene may influence response to lomitapide. This study further presented a number of variants that may be uniquely associated with higher or lower response to lomitapide treatment. Further research into the effect of *MTTP* variants on response to lomitapide is warranted.

## Supplementary Information

Below is the link to the electronic supplementary material.

Supplementary Material 1

## Data Availability

The datasets used and/or analyzed during the current study are available from the corresponding author on reasonable request.
