# Supplementary material for: Lomitapide response in a cohort of patients with homozygous familial hypercholesterolemia and the potential influence of MTTP gene variants
Source: Orphanet J Rare Dis. 2025 Oct 14;20:513. doi: 10.1186/s13023-025-04033-3 (PMC12522312; doi:10.1186/s13023-025-04033-3)
Supplement: Supplementary file 1 — Supplementary Material 1 [file 13023_2025_4033_MOESM1_ESM.docx]

# Additional File 1

**Supplementary Table 1.** Concomitant medications other than lipid-lowering therapy prescribed to patients during lomitapide treatment

| Patient ID | Therapies |
| --- | --- |
| **9** | Prasugrel 10 mg  Acetylsalicylic acid 100 mg  Metoprolol 50 mg  Amiodarone 100 mg  Amlodipine 10 mg  Valsartan 160 mg  Hydrochlorothiazide 25 mg  Pantoprazole 40 mg |
| **11** | Acenocoumarol 4 mg  Metoprolol 50 mg |
| **6** | Acetylsalicylic acid 100 mg |
| **5** | Acetylsalicylic acid 100 mg  Metoprolol 50 mg |
| **13** | Acetylsalicylic acid 100 mg  Metoprolol 50 mg  Valsartan 160 mg  Hydrochlorothiazide 25 mg  Pantoprazole 40 mg |
| **7** | Acetylsalicylic acid 100 mg  Metoprolol 50 mg |
| **3** | Acetylsalicylic acid 100 mg  Metoprolol 50 mg  Valsartan 160 mg |
| **4** | Acetylsalicylic acid 100 mg  Metoprolol 50 mg  Valsartan 80 mg  Hydrochlorothiazide 12.5 mg  Pantoprazole 40 mg |
| **1** | Metoprolol 50 mg |
| **10** | None |
| **2** | Acetylsalicylic acid 100 mg |
| **8** | None |
| **12** | Acetylsalicylic acid 100 mg  Valsartan 160 mg  Hydrochlorothiazide 25 mg  Pantoprazole 40 mg |
